# Combination of single-nucleus and bulk RNA-seq reveals the molecular mechanism of thalamus haemorrhage-induced central poststroke pain

**DOI:** 10.3389/fimmu.2023.1174008

**Published:** 2023-04-20

**Authors:** Tianfeng Huang, Yinggang Xiao, Yang Zhang, Yali Ge, Ju Gao

**Affiliations:** ^1^ Northern Jiangsu People’s Hospital Affiliated to Yangzhou University/Clinical Medical College, Yangzhou University, Yangzhou, Jiangsu, China; ^2^ Yangzhou Key Laboratory of Anesthesiology, Northern Jiangsu People’s Hospital, Yangzhou, Jiangsu, China

**Keywords:** thalamic haemorrhage, central poststroke pain, single-nucleus RNA sequencing, bioinformatics analysis, cell−cell communication

## Abstract

Central poststroke pain (CPSP) induced by thalamic haemorrhage (TH) can be continuous or intermittent and is accompanied by paresthesia, which seriously affects patient quality of life. Advanced insights into CPSP mechanisms and therapeutic strategies require a deeper understanding of the molecular processes of the thalamus. Here, using single-nucleus RNA sequencing (snRNA-seq), we sequenced the transcriptomes of 32332 brain cells, which revealed a total of four major cell types within the four thalamic samples from mice. Compared with the control group, the experimental group possessed the higher sensitivity to mechanical, thermal, and cold stimuli, and increased microglia numbers and decreased neuron numbers. We analysed a collection of differentially expressed genes and neuronal marker genes obtained from bulk RNA sequencing (bulk RNA-seq) data and found that Apoe, Abca1, and Hexb were key genes verified by immunofluorescence (IF). Immune infiltration analysis found that these key genes were closely related to macrophages, T cells, related chemokines, immune stimulators and receptors. Gene Ontology (GO) enrichment analysis also showed that the key genes were enriched in biological processes such as protein export from nucleus and protein sumoylation. In summary, using large-scale snRNA-seq, we have defined the transcriptional and cellular diversity in the brain after TH. Our identification of discrete cell types and differentially expressed genes within the thalamus can facilitate the development of new CPSP therapeutics.

## 1 Introduction

Currently, stroke is one of the most prevalent diseases threatening human life and health worldwide, with a very high disability rate and a high mortality rate; its treatment cost is high, and it places a heavy burden on people’s lives (1). The prevalence of stroke in China is on the rise. global burden of disease study (GBD; http://ghdx.healthdata.org/) data showed that the prevalence of hemorrhagic stroke in China in 2019 was 306/100,000 (age-standardized prevalence 215/100,000). According to the China Health Statistical Yearbook 2019 (http://www.nhcgov.cn/), the crude death rate of stroke in China in 2018 was 160 per 100,000 for rural residents and 129 per 100,000 for urban residents. There are two types of stroke: ischaemic stroke and haemorrhagic stroke. Although ischaemic stroke is more common than haemorrhagic stroke, haemorrhagic stroke has a higher mortality and disability rate than ischaemic stroke. Central poststroke pain (CPSP) is a neuropathic pain syndrome caused by damage to the spinothalamic pathway after stroke, which usually occurs within 6 months after haemorrhagic or ischaemic stroke (2). CPSP can be continuous or intermittent and is accompanied by paresthesia, and long-term pain can also lead to emotional changes such as anxiety and depression in patients, which further aggravate pain and seriously affect the quality of life of patients (3, 4). Thalamic haemorrhage is the main cause of CPSP, and the sites of thalamic haemorrhage with a high incidence of CPSP mainly include the ventral posterolateral/ventral posteromedial nuclei of the thalamus (VPL/VPM) (5, 6).

As research has progressed and more evidence has been collected, many theories concerning the pathogenesis of CPSP have emerged, mainly including the disinhibition theory, central sensitization, neuroplasticity, changes in spinothalamic conduction pathways, infiltration of inflammatory cells, and activation of glial cells (5, 7–9). An increasing number of studies have shown that the dynamic changes in gene regulation at the injury site after thalamic haemorrhage may be a key factor leading to the occurrence of CPSP. For example, after thalamic haemorrhage, m6A-modified RNA demethylase FTO stabilizes TLR mRNA in neurons, leading to an increase in the expression levels of the TLR4 protein and causing central sensitization, which is involved in the occurrence and development of CPSP (10). In addition to neurons, other cell types in the brain also play an important role in the regulation of CPSP. After thalamic haemorrhage, both microglia and astrocytes are stimulated and activated by the injury, and the nonreceptor tyrosine kinase Fgr expressed in microglial cells mediates the development of CPSP by activating the NF-κB signalling pathway and causing CNS inflammation (11). In short, the mechanism of CPSP is intricate, and thus far, CPSP is still difficult to cure. Clinically, drugs for the treatment of CPSP are usually addictive or not very effective, and long-term use of several CPSP drugs may cause serious side effects (12). Therefore, it is necessary to further explore the underlying mechanisms of CPSP occurrence and development at the molecular and cellular levels.

Several bulk RNA-sequencing (bulk RNA-seq) studies using brain tissue have shown that cerebral haemorrhage can lead to changes in multiple genes (13, 14). However, bulk RNA-seq technology can only provide the overall transcriptional level change, which is the analysis of thousands or even millions of large samples of cells, the average result of a large number of cell sequencing analyses, or the reflection of a dominant number of cell data, all of which mask important information and ignore the heterogeneity between cells or cell subtypes. This method is not conducive to the understanding and research of cellular biodiversity, so more accurate sequencing methods are needed. In recent years, single-cell RNA sequencing (scRNA-seq) and single-nucleus RNA sequencing (snRNA-seq) have been used to obtain the transcriptional expression profile of certain cell types at the molecular level. Through functional analysis, this gene expression information can be linked with cell function, and the use of spatial mapping can locate cells in a certain tissue or organ and finally obtain a map of different types of cells (15, 16). Therefore, using scRNA-seq and snRNA-seq, especially snRNA-seq, on brain tissue is becoming increasingly widespread in neuroscience research, mainly because brain tissue cells are not easy to separate (16, 17). However, to date, there has been no research on the distribution of cell types in thalamic tissue and the changes in the transcriptome in a single cell after the occurrence of CPSP caused by thalamic haemorrhage. Therefore, to understand the cellular and molecular mechanisms of CPSP caused by thalamic haemorrhage in detail and provide a theoretical basis for the clinical treatment of CPSP, the snRNA-seq research method is essential.

In this study, for the first time, we used snRNA-seq and bulk RNA-seq to measure mRNA expression in the thalami of thalamic haemorrhage model mice. Through differential gene analysis and cell cluster analysis, the specific cells and their key genes that affect the progression of thalamic haemorrhage were identified, and the intercellular interaction in thalamic haemorrhage was revealed for the first time. We further explored the underlying mechanism of CPSP occurrence and development at the molecular and cellular levels.

## 2 Materials and methods

### 2.1 Animals

Adult C57BL6 mice (age, ~7-8 weeks, weight, ~25-30 g) were purchased from Beijing Weitong Lihua Experimental Animal Technical Co., Ltd., and kept under a 12-h light/dark cycle in the same colony room, with controlled temperature (23 ± 1°C) and humidity (50 ± 5%). By a random number table, the animals were assigned into the following two groups: (1) NS (n = 15): mice that received normal saline (NS). (2) COL (n = 15): mice that received collagenase IV (Coll IV). The brain tissue of every three mice in the same group constituted one sample, three samples were selected from each of the two groups for bulk RNA-seq, and snRNA-seq was performed for the remaining two samples in each groups. The research protocol and animal experiments were approved by the Animal Care and Use Committee of Wuhan Servicebio Biotechnology Co., Ltd. (Wuhan, China; approval no. 2022045).

### 2.2 Thalamic haemorrhage−induced CPSP model

Modelling was performed according to our previous article (18). Isoflurane was administered to anaesthetize the mice, which were then placed in a stereotactic frame. Using a glass micropipette, Coll IV (0.01 U/10 nl, dissolved in saline solution; Sigma−Aldrich; Merck KGaA) was injected into the right VPM and VPL nuclei of the thalamus. The NC group was injected with 10 nl of sterile physiological saline. Following administration, the glass micropipette was held in position for 10 min to enable the Coll IV to fully disperse, and then the glass micropipette was slowly removed. Then, iodophor and sterile saline were used to sterilize the surgical area, which was later stitched with a wound clip.

### 2.3 Behavioural tests

This experiment used pain behaviour tests to measure the sensitivity of mice to mechanical, thermal, and cold stimuli. Testing was performed every hour. First, the mechanical sensitivity test was conducted by placing the mouse in a plexiglass chamber with an elevated grid floor and stimulating the hind limbs with two calibrated von Frey filaments (calibration values of 0.07 and 0.4 g, Stoelting, USA) to record the number of paw withdrawals as the response frequency [(number of withdrawals/10 trials) × 100% = response frequency]. Then, the thermal sensitivity test was performed using the same method, and a Model 336 analgesia metre measured the response time of mice to harmful heat stimulation. Each test was repeated 5 times with a 5-minute interval, and the cut-off time was set at 20 seconds to avoid tissue damage. Finally, the cold sensitivity test was conducted by placing the mouse on a plate with temperature monitoring in a plexiglass chamber and recording the response time of the mouse to harmful cold stimulation. The cut-off time was set at 20 seconds to avoid tissue damage.

### 2.4 Date collection

Seven days after the intervention, injured thalamic tissue was collected for sequencing. Wuhan Servicebio Biotechnology Co., Ltd. performed bulk transcriptome sequencing, and Hangzhou Lianchuan Biotechnology Co., Ltd. conducted snRNA-seq using four randomly analysed samples from twelve mice.

### 2.5 Single nucleus preparation

Brain tissue (0.1-0.3 g) from each group was washed with 1x PBS (Thermo Fisher, USA), minced and homogenized in a glass homogenizer containing 3 mL of Buffer A (250 mM sucrose (Sangong, China), 10 mg/mL bovine serum albumin (BSA, Thermo Fisher), 5 mM MgCl_2_ (Sinopharm, China), 0.12 U/μL RNasin (Promega, USA), 0.06 U/mL SUPERasenTM RNase Inhibitor (Thermo Fisher), 1x Protease Inhibitor (CST, USA), and mixed 10 times with both large and small pestles on ice. The mixture was filtered through a 100 µm cell sieve filter, and the filter was washed twice with 1750 mL of Buffer A. After the addition of Triton X-100 (Thermo Fisher), a nonionic surfactant with a concentration of 0.5%, the mixture was further homogenized with a pestle and mixed 50 times. After the mixture passed through a 35 µm cell sieve membrane, the following steps were performed in sequence: the mixture was centrifuged at 500 g at 4°C for 5 min, the supernatant was discarded, and the pellet was resuspended in 1 mL of citric acid solution (0.25 M sucrose (Sangong), 25 mM citric acid (Sigma), 1 µg/mL of Hoechst 33342 (Thermo Fisher)), the mixture was centrifuged at 500 g at 4°C for 5 min, the supernatant was discarded, the pellet was resuspended in 1 mL Buffer B, and the mixture was centrifuged at 3000 g at 4°C for 5 min, the supernatant was discarded, and the pellet was resuspended in 200 µL Buffer B (320 mM sucrose (Sangon), 10 mg/mL BSA (Thermo Fisher), 3 mM CaCl_2_ (Sinopharm), 2 mM MgAc_2_ (Sinopharm), 10 mM Tris-HCl (Sigma), 0.1 mM EDTA (Sigma, USA), 1 mM DTT (Invitrogen, USA), 1 mM DTT (Invitrogen), 0.12U/μL RNasin (Promega), 0.06U/mL SUPERasenTM RNase Inhibitor (Thermo Fisher), 1xProtease Inhibitor (CST)), and centrifuged at 20000 × g for 3 min at 4°C. Finally, the pellet was stained with trypan blue (Thermo Fisher) and the nuclei were counted by CountStar.

### 2.6 snRNA-seq and data processing

Single nuclei were run on a 10× Chromium system (10 × Genomics) and then subjected to library preparation by LC Sciences, following the recommended protocol for the Chromium Single Cell 30 Reagent Kit (v2, Chemistry). Libraries were run on the HiSeq4000 instrument for Illumina sequencing. Sequencing results were demultiplexed and converted to FASTQ format using Illumina bcl2fastq software (v2.20). Sample demultiplexing, barcode processing and single-cell 3’ gene counting were performed using a 10× Cell Ranger package (v1.2.0; 10 × Genomics). Reads were aligned to the mm10 reference assembly (v1.2.0; 10 × Genomics). The snRNA-seq data (Cellranger_result) contained 4 samples (C1, C2, M1, M2).

The expression profile was read using the Seurat package, and the low expression genes were screened out (nFeature_RNA > 50 & percent.mt < 5). Normalization, homogenization, principal component analysis (PCA) and uniform manifold approximation and projection (UMAP) analysis were successively carried out on the data. The optimal number of principal components (PCs) was observed by ElbowPlot, and the position relationship between each cluster was obtained by T-distributed stochastic neighbour embedding (tSNE) analysis. The cluster of cells that were important to the occurrence of the disease was annotated by the celldex package (19). Finally, we extracted the marker genes of each cell subtype from the single-cell expression profile by the FindAllMarkers function with the parameter (logfc.threshold = 1). With the criteria of p_val_adj ≤ 0.05 and |avg_log2FC| ≥ 1, the genes were screened as unique marker genes in each cell subtype.

### 2.7 Ligand−receptor interaction analysis

CellChat, a tool that enables quantitative inference and analysis of intercellular communication networks from single-cell data, uses network analysis and pattern recognition methods to predict the main signal inputs and outputs of cells and how these cells and signals result in specific functions (20). In this analysis, standardized single-cell expression profiles were used as input data, and cell subtypes obtained from single-cell analysis were used as cell information which was visualized as a network graph. Cell-related interactions were analysed, and the intensity (weights) and frequency (counts) of interactions between cells were used to quantify the closeness of interactions to observe the activity degree and influence of each type of cell in the disease.

### 2.8 Key gene identification

We used RNA-seq data to further identify the key genes involved in thalamic haemorrhage among the candidate genes. Differential analysis was carried out in the two groups of samples by the limma package (21) to identify differentially expressed genes in the two groups of samples, and the screening criteria of differentially expressed genes was |LogFC| > 0.585 and p <0.05. Then, the differentially expressed genes and candidate genes (neurons) were intersected to explore potential key genes. The R package clusterProfiler was used to comprehensively explore the functional correlation of these key genes. GO and KEGG were used for the evaluation of relevant functional categories. GO and KEGG enriched pathways with both p values and q-values less than 0.05 were considered significant pathways.

### 2.9 Immune infiltration assay

The immune microenvironment is mainly composed of immune-related fibroblasts, immune cells, extracellular matrix, various growth factors, inflammatory factors and special physicochemical characteristics (22, 23). It substantially affects the diagnosis, survival outcome and clinical severity of many diseases. The CIBERSORT method is a widely used method for evaluating immune cell types in microenvironments (24). Based on the principle of support vector regression, deconvolution analysis was performed on the expression matrix of immune cell subtypes in this method. The expression matrix contains 547 biomarkers that distinguish 25 mouse immune cell phenotypes, including T cells, B cells, plasma cells, and myeloid cell subsets. In this study, the CIBERSORT algorithm was used to analyse the sample data, which was used to infer the relative proportion of 25 kinds of immunoinfiltrating cells, and Spearman correlation analysis was conducted to examine the correlations between gene expression and immune cell type. The sum of all estimated immune cell type scores in each sample was equal to 1, and the difference in immune cell content was tested by t-test. The correlations between these key genes and different immune factors were then validated using the TISIDB database (http://cis.hku.hk/TISIDB/) (25).

### 2.10 Gene set enrichment analysis

GSEA uses a predefined gene set to sort the genes according to the degree of differential expression in the two sample groups and then evaluates whether the preset gene set is enriched at the top or bottom of the sorting table (26). In this study, GSEA was used to compare the differences in signalling pathways between the high expression group and the low expression group and to explore the molecular mechanism of key genes in the two sample groups. The number of permutations was set to 1000, and the permutation type was set to phenotype. In addition, we performed reverse prediction of related miRNAs *via* the TargetScanMouse database (https://www.targetscan.org/mmu_72/) and visualization *via* Cytoscape (v3.9.1) for the key genes (27, 28).

### 2.11 Genome-wide association studies

The Gene Atlas database (http://geneatlas.roslin.ed.ac.uk/) is a large database that documents associations between hundreds of traits and millions of variants using the UK Biobank cohort (29). These associations were calculated using 452,264 UK individuals in the UK Biobank database, covering a total of 778 phenotypes and 30 million loci. According to trait and region information or gene options in the Gene Atlas database, the thalamic haemorrhage phenotypes were searched to determine the chromosomal pathogenic sites of the key genes associated with thalamic haemorrhage.

### 2.12 Regulatory network analysis of key genes

In this study, the R package “RcisTarget” was used to predict transcription factors (30). All calculations performed by RcisTarget are based on motifs. The normalized enrichment score (NES) of a motif depends on the total number of motifs in the database. In addition to the motifs annotated by the source data, further annotation files were inferred based on motif similarity and gene sequences. The first step in estimating the overexpression of each motif across a gene set is to calculate the area under the curve (AUC) for each motif-motif-set pair (31). This calculation was performed based on the recovery curve calculation of the gene set versus motif ordering. The NES of each motif was calculated based on the AUC distribution of all motifs in the gene set. We used MM9-500 bp-upgrade-10species.mc9NR for the gene-motif rankings database.

### 2.13 Immunofluorescence of key genes

After animals were deeply anesthetized with isoflurane, they were perfused with 100-300 ml of 4% paraformaldehyde in 0.1 M phosphate buffer (pH 7.4). The brain was harvested, postfixed at 4°C for 24 h, and cryoprotected in 30% sucrose overnight. The tissues were sectioned at the thickness of 30 mm on a cryostat. After being blocked with PBS containing 5% goat serum and 0.3% Triton X-100 for 1 h at 37°C, the sections were incubated overnight at 4°C with rabbit anti-Apoe(CST, 1:100, catalog number: 49285) or rabbit anti-Abca1(Novus Biologicals, USA 1:200, catalog number: NB48-105) or rabbit anti-Hexb(Thermo Fisher, 1:100, catalog number: PA5-101082). The sections were then incubated with goat anti-rabbit IgG conjugated with Cy2 (Jackson ImmunoResearch, USA, 1:500, catalog number:111-225-144) for 1 h at room temperature. Control experiments included omission of the primary antiserum and substitution of normal rabbit serum for the primary antiserum. The sections were finally mounted using VectaMount permanent mounting medium (Vector Laboratories, USA) or Vectashield plus 40, 6-diamidino-2-phenylindole (DAPI) mounting medium (Vector Laboratories). All images were observed using a Leica DMI4000 fluorescence microscope and captured with a DFC365FX camera (Leica, Germany). Positive cells were calculated manually.

### 2.14 Statistical analysis

R language software (v4.0) was used for statistical analysis of our data. Behavioral and IF results were analyzed using two independent sample t-tests. All statistical tests were two-sided, and P < 0.05 was considered statistically significant.

## 3 Results

### 3.1 Thalamic haemorrhage-induced CPSP

The mice with thalamus haemorrhage displayed persistent and intense mechanical pain abnormalities, thermal hyperalgesia, and abnormal cold pain on the contralateral side of their bodies. When Coll IV was microinjected, there was a significant increase in the frequency of claw retraction on the contralateral side in response to 0.07 g and 0.4 g von Frey wires, along with a significant decrease in the latency of claw retraction in response to thermal and cold stimulation. These pain hypersensitivity reactions occurred 1 day after microinjection and lasted for at least 7 days. In contrast, microinjection of saline did not significantly alter the retraction frequency and latency of the contralateral basal claw. Coll IV and saline microinjections did not affect the frequency or latency of ipsilateral basal claw retraction (**Supplementary Figure 1**).

### 3.2 Single-cell level analysis of cell ranger result data

**Figure 1:**
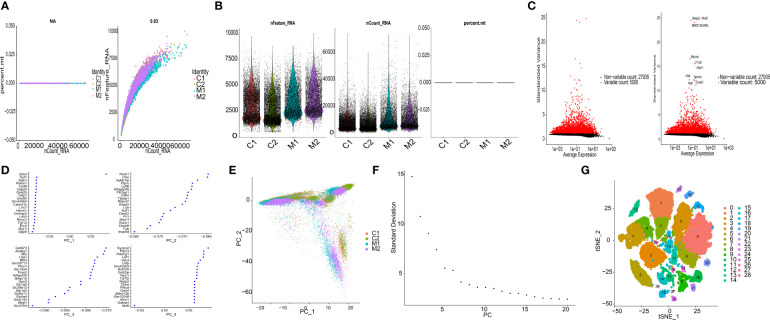
Characterization of thalamic haemorrhage by snRNA-seq. **(A)** The left figure shows the relationship between cell sequencing depth and mitochondrial content, the right figure shows the relationship between sequencing depth and gene quantity, and the two are positively correlated. **(B)** Single-cell quality control, showing cell count, gene count, and sequencing depth per sample. **(C)** Variance plots of genes and traits with significant differences between cells. **(D)** PCA presentation. **(E)** Distribution of PCs. Dots represent cells, and colours represent samples. **(F)** Variance ranking plot for each PC. **(G)** According to the important components available in PCA, cells are divided into 29 clusters by the tSNE algorithm.

snRNA-seq data (GSE227003) contained 4 samples (C1, C2, M1, M2). Primary assessment with 10× Cell Ranger for the NS group (C1, C2) reported 9681 and 10438 cell barcodes with 3026 and 2233 median genes per cell sequenced to 45.4% and 47.8% sequencing saturation with 40780 and 37953 mean reads per cell, respectively. Primary assessment with this software for the COL group (M1, M2) reported 5943 and 6270 cell barcodes with 3270 and 3460 median genes per cell sequenced to 53.3% and 46.7% sequencing saturation with 67365 and 63897 mean reads per cell. The main details of the sequencing are shown in **Figures 1A, B**.

In this analysis, the data samples were initially screened through nFeature_RNA and nCount_RNA, and the 10 genes with the highest standard deviation are displayed (**Figure 1C**). We performed PCA dimensionality reduction analysis on 20 of the genes and found that they had different scoring values in different dimensions (**Figure 1D**). However, PCA dimension reduction analysis among samples found that the overall difference between samples was not obvious, and the optimal PC number observed by ElbowPlot was 15 (**Figures 1E, F**). Finally, the tSNE algorithm was used to cluster cells, and all cells were clustered into 29 cell subsets (**Figure 1G**).

### 3.3 Annotation of cell subpopulations from snRNA-seq data

**Figure 2:**
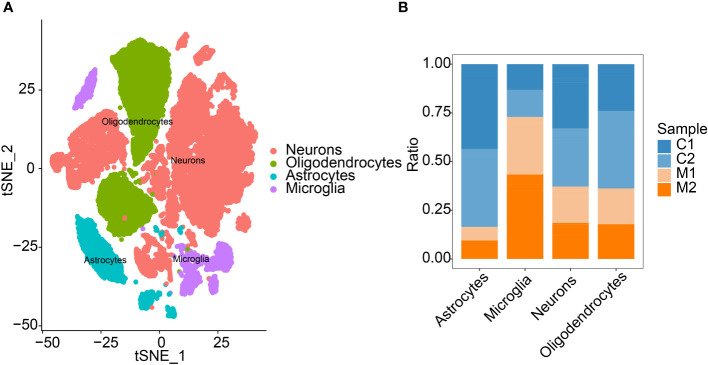
Annotation of cells. **(A)** Cell annotation of 29 clusters. Twenty-nine clusters were annotated into 4 cell types. **(B)** Differences in the proportions of the 4 types of cells in the two sample groups.

In this study, each subtype was annotated by the R package SingleR, and 29 clusters were annotated into the 4 cell categories of neurons, oligodendrocytes, astrocytes and microglia (**Figure 2A**). The number of immune cells and microglia increased in the COL group (**Figure 2B**). Finally, we extracted the marker genes unique to each cell subtype from the single-cell data through the FindAllMarkers function (**Appendix 1**).

### 3.4 Cell-to-cell communication analysis

**Figure 3:**
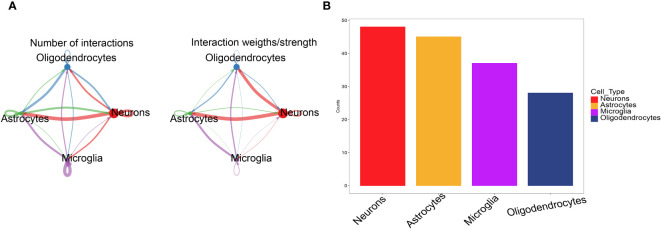
CellChat evaluates cell communication. **(A)** The cell interaction network between 4 types of cells. The edge width indicates the probability and strength of communication between cells. **(B)** Comparison of the total number of interactions in the communication network between the 4 types of cells, decreasing from left to right, the strongest being neurons.

We used the software package CellChat to analyse the ligand−receptor relationships in the single-cell expression profile. Next, we found complex pairs of interactions between these cell subtypes (**Figure 3A**). Finally, we statistically found that cells such as neurons and astrocytes have more potential interactions with other cells (**Figure 3B**). Therefore, neuron marker genes were ultimately selected as the candidate gene set.

### 3.5 Screening of key genes related to cell communication

**Figure 4:**
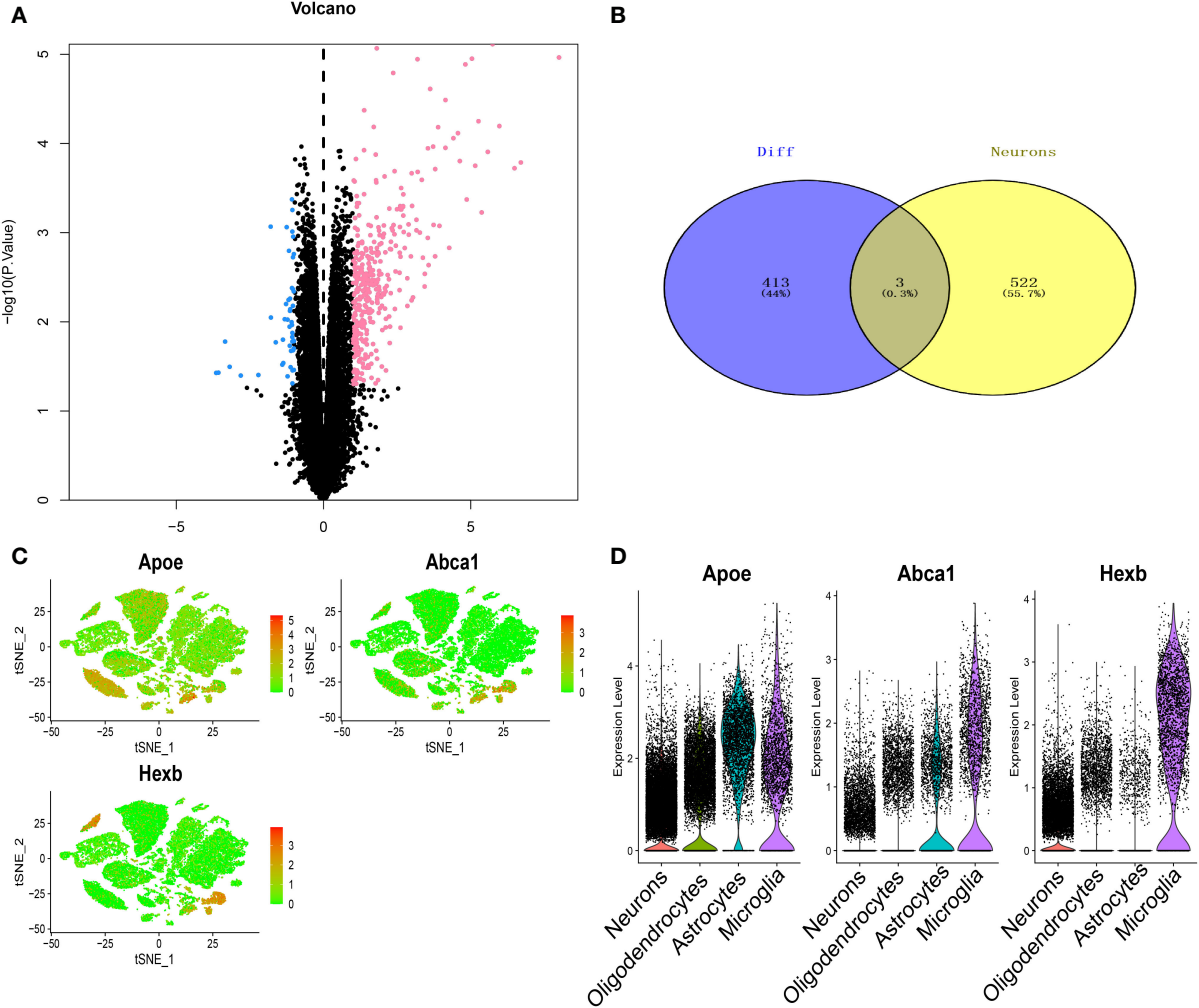
Screening of key genes in thalamic haemorrhage. **(A)** Volcano plot of differentially expressed genes by RNA-seq. Pink indicates upregulation of gene expression, and blue indicates downregulation of gene expression. **(B)** Venn diagram of differentially expressed genes and neuron markers. **(C)** Key gene t-SNE dimensionality reduction analysis. **(D)** Expression profiles of key genes in single cells.

RNA-seq data were analysed by the limma package, and a total of 416 differentially expressed genes were obtained, of which 367 were upregulated and 49 were downregulated (**Figures 4A, B**). Three key genes, Apoe, Abca1 and Hexb, were identified after the intersection of differentially expressed genes and the candidate neuron marker gene set (**Figure 4C**). The expression of these three key genes in the four cell types is shown in **Figure 4D**. We further performed pathway analysis on the three genes (**Supplementary Figure 2**). GO enrichment analysis showed that these key genes were mainly enriched in the lipid localization pathway. KEGG enrichment analysis revealed that these key genes were mainly enriched in pathways such as cholesterol metabolism pathway.

### 3.6 Analyses of the immune microenvironment

**Figure 5:**
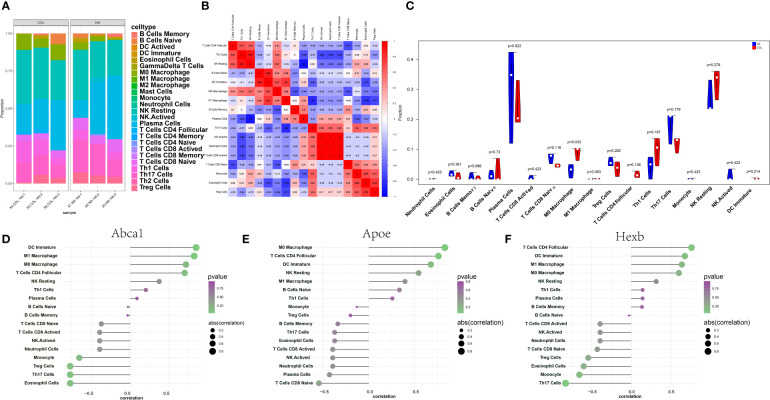
Immune infiltration in thalamic haemorrhage. **(A)** Relative percentages of 25 immune cell subsets. **(B)** Pearson correlation between 25 kinds of immune cells. Blue indicates a positive correlation, and red indicates a positive correlation. **(C)** The difference in immune cell content between control and disease samples, with control samples in blue and disease samples in red. **(D–F)** Pearson correlation between key genes Abca1, Apoe and Hexb and immune cells, positive correlation on the right and negative correlation on the left.

Through the analysis of the relationship between key genes and immune infiltration in the RNA-seq dataset, the effect of key genes on the progression of thalamic haemorrhage was explored. The immune cell content of each sample is shown (**Figure 5A**), and there are several significant correlations between the key genes and the levels of immune infiltration (**Figure 5B**). In addition, compared with the NS sample, the COL sample had a significantly higher M0 macrophage level (**Figure 5C**). After exploring the relationship between the key genes and immune cells, it was found that each key genes was highly correlated with immune cells. Abca1 was positively correlated with immature dendritic cells (DCs) and M1 macrophages and negatively correlated with eosinophils and Th17 cells (**Figure 5D**). Apoe was positively correlated with M0 macrophages and CD4+ follicular T cells and negatively correlated with CD8+ naive T cells and plasma cells (**Figure 5E**). Hexb was positively correlated with CD4+ follicular T cells and immature DCs and negatively correlated with Th17 cells and monocytes (**Figure 5F**). These correlations were verified using the TISIDB database and suggest that the key genes are mainly related to immunosuppressants, chemokines, immunostimulants, receptors and major histocompatibility complex (MHC) proteins (**Supplementary Figure 3**).

### 3.7 CPSP key genes signalling pathway enrichment analysis

**Figure 6:**
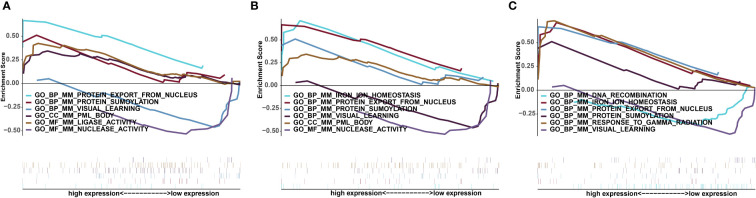
Significantly enriched GSEA pathways for key genes. **(A)** Apoe. **(B)** Abca1. **(C)** Hexb. The upper part of the abscissa indicates high expression, and the lower part indicates low expression.

Next, we studied the specific signalling pathways enriched in the three key genes and explored the underlying molecular mechanisms by which these key genes affect disease progression. The GSEA results showed that the highly expressed Apoe gene was enriched in the biological processes protein export from nucleus and protein sumoylation, the cellular component promyelocytic leukaemia (PML) body and the molecular function ligase activity (**Figure 6A**). The highly expressed Abca1 gene was enriched in the biological processes iron ion homeostasis, protein export from nucleus, and protein sumoylation and the cellular component PML body (**Figure 6B**). The highly expressed with Hexb gene was enriched in the biological processes iron ion homeostasis, protein export from nucleus, protein sumoylation and response to gamma radiation (**Figure 6C**).

### 3.8 Analysis of key gene-related transcription regulation

We applied the three key genes to the candidate gene set in this analysis and found that they are regulated by common mechanisms such as multiple transcription factors. Therefore, enrichment analysis was performed on these transcription factors using cumulative recovery curves (**Supplementary Figures 4A, B**). The motif annotation with the highest AUC was jaspar:MA1091.1, and the three key genes were enriched in this motif, namely, Abca1, Apoe and Hexb, and the normalized enrichment score (NES) was 7.32. In addition, we displayed all the enriched motifs and corresponding transcription factors of the key genes. The three key genes were reverse predicted by the TargetScanMouse database and yielded 99 miRNAs and a total of 103 mRNA−miRNA relationship pairs, which were visualized using Cytoscape (**Supplementary Figure 4C**).

### 3.9 GWAS analysis of the key genes

**Figure 7:**
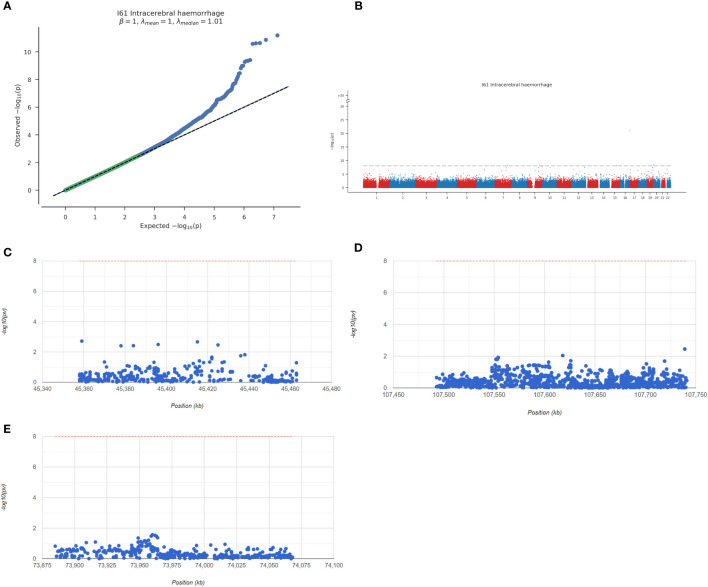
Overview of key genes analysed by GWAS. **(A)** Q-Q diagram. GWAS data can identify SNP sites with significant associations. **(B)** Manhattan map representing meta-GWAS results. **(C–E)** Chromosomal pathogenic regions of key genes.

Next, we analysed the GWAS data of the disease to confirm the pathogenic regions of the 3 key genes. As shown in **Figure 7A**, the Q-Q chart shows significant disease-associated single nucleotide polymorphism (SNP) loci identified by GWAS data. The key SNP sites distributed in the enrichment area were found by using the precise location identified by GWAS data (**Figure 7B**). The pathogenic region of SNPs corresponding to Apoe, Abca1 and Hexb is shown, in which Apoe is located in the pathogenic region of chromosome 19, Abca1 is located in the pathogenic region of chromosome 9, and Hexb is located in the pathogenic region of chromosome 5 (**Figures 7C–E**). The significant SNP loci corresponding to the 3 genes are shown in **Appendix 2**.

### 3.10 Correlation analysis between key genes and disease regulatory genes

We obtained the genes associated with thalamic haemorrhage through the GeneCards database (https://www.genecards.org/) (32). We analysed the expression levels of the three key genes and the thalamic haemorrhage-related genes and found that the expression levels of Cp, Notch3, and Plat were different between the two groups of samples (**Supplementary Figure 5A**). Among them, Apoe was significantly negatively correlated with Krit1 (Pearson r=-0.85), and Hexb was significantly positively correlated with Cst3 (Pearson r=0.99) (**Supplementary Figure 5B**).

### 3.11 Apoe, Abca1 and Hebx are increased after CPSP

**Figure 8:**
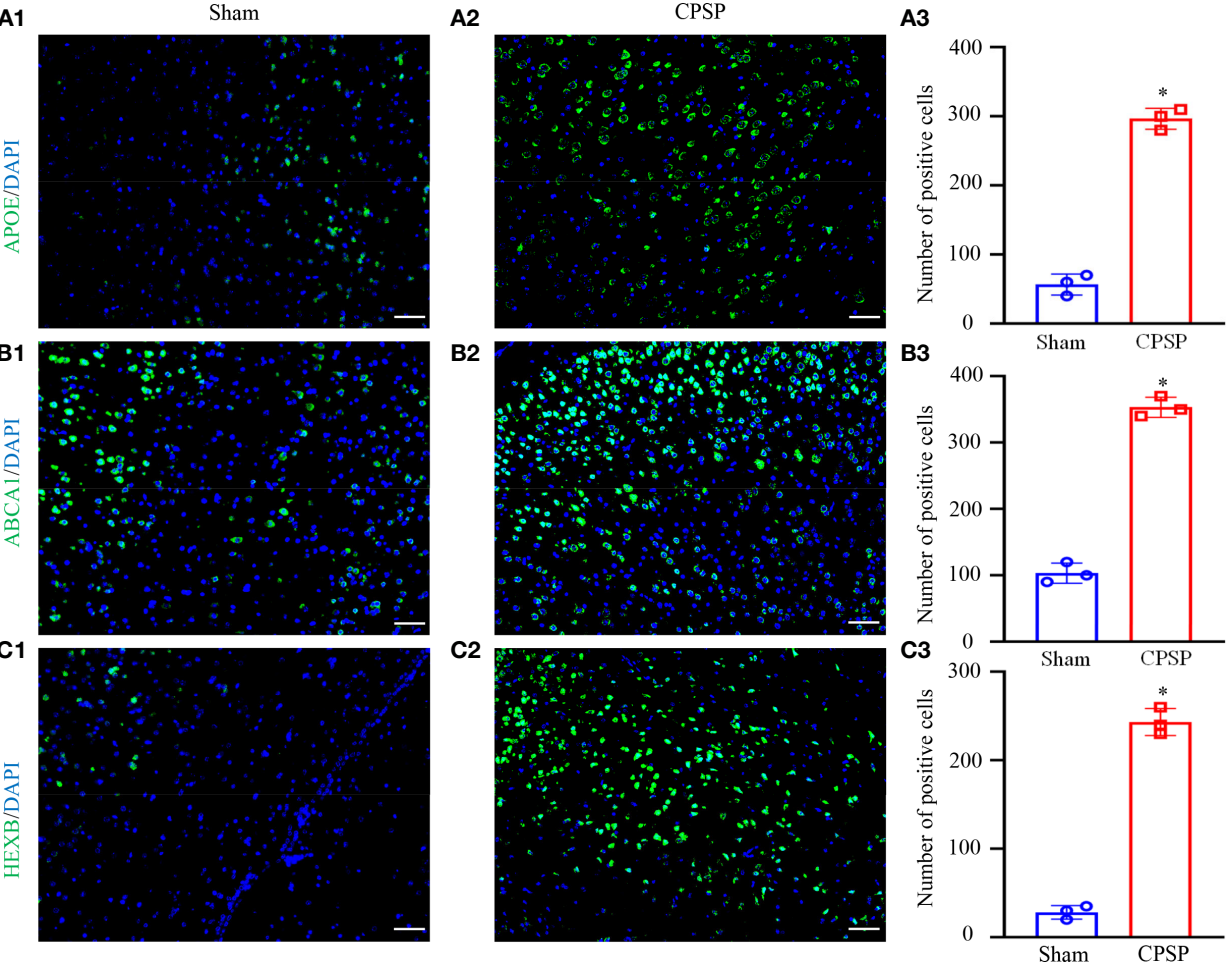
IF result of the key genes. **(A)** Apoe/DAPI. **(B)** Abca1/DAPI. **(C)** Hexb/DAPI. The pictures from left to right are the sham operation group, the control group and the number of positive cells. All images were taken at 20× magnification. Scale bar=50 μm. *P < 0.05 versus Sham group.

We examined whether Apoe, Abca1 and Hebx are altered in thalamus. The number of Apoe labeled cells in this thalamic region on day 7 post-Coll IV microinjection was increased by 5.29-fold as compared with that after saline microinjection (**Figures 8A1-A3**). The number of Abca11 labeled cells in this thalamic region on day 7 post-Coll IV microinjection was increased by 3.43-fold as compared with that after saline microinjection (**Figures 8B1-B3**). The number of Hexb labeled cells in this thalamic region on day 7 post-Coll IV microinjection was increased by 8.68-fold as compared with that after saline microinjection (**Figures 8C1-C3**).

## 4 Discussion

Our study collected thalamic tissue from 15 normal mice and 15 CPSP model mice for snRNA-seq and bulk RNA-seq, which significantly expanded the newly emerging and referential thalamic cell atlas and provided insights into the relationship between the thalamus and the occurrence and development of CPSP disease. By combining sn/bulk RNA-seq data with cell communication and transcription factor regulation analysis, we provide a detailed overview of the thalamic cell library and related molecular tags. We quantified each cell and highlighted the specific characteristics of CPSP thalamic cells. In each cell and subtype, we identified specific marker genes, and further screened, verified, and analysed the key neuronal genes with the richest cell communication in the development of CPSP. Our results begin to reveal the molecular basis of the pathophysiology of CPSP and the cellular response.

As the most important cell for brain function, neurons transmit signals and exchange information to other cells through synapses and cell membranes and are the cells with the most frequent cell communication among all brain cells (33, 34). Astrocytes, the most abundant cells in the brain, are neuroepithelial-derived cells that, by forming the blood−brain barrier, providing nutritional support to neurons and forming tripartite synapses with neurons, play an important role in regulating neurovascular function necessary for brain activity (35, 36). Microglia, as macrophages that colonize the central nervous system, play an important role in the immune response and reduce brain tissue damage by mediating inflammation, immune surveillance, polarization, and phagocytosis of cellular debris (37–39). Our snRNA-seq data and cellular interaction network results also support the above point. Thalamic haemorrhage leads to changes in the cell composition ratio, a significant decrease in the number of neurons and astrocytes, and an increase in the number of microglia, which play a role in clearing damaged tissue and promoting functional recovery.

Neurons are the key cells involved in cell communication during thalamic haemorrhage (TH) and neuralgia, and their function is tied to their molecular composition. ApoE is an important component of lipoproteins in the peripheral and central nervous systems and participates in the regulation of lipid transport, lipid metabolism and cholesterol balance in the body. Abnormal lipid metabolism and elevated lipid levels caused by the abnormal expression of ApoE are closely related to the occurrence and progression of cerebral haemorrhage (40–42). Abca1 is a key player in the reverse cholesterol transport pathway, and its expression affects neuroinflammation and neuronal degeneration and death (43–45). Hexb is an essential component of the assembly of hexosaminidases involved in lysosomal glycolipid degradation/processing and has been implicated in motor neuron disease and gangliosidosis (46–48). We have proven for the first time that Apoe, Abca1 and Hexb are the key genes expressed by neurons that affect the development of CPSP.

We also found that they are closely related to the level of immune cell infiltration, play an important role in the immune microenvironment and are closely related to the transport of proteins from the nucleus to the cytoplasm. Previous studies have demonstrated that the three key genes have significant effects on immune function, which supports our hypothesis that immune infiltration may be a crucial pathway for these key genes to mediate CPSP. For instance, APOE has been shown to affect the progression of Alzheimer’s disease through immune regulation. Its high expression has been linked to the activation of cytotoxic T lymphocyte (CTL) responses and inhibition of cancer growth (49, 50). Moreover, studies have revealed that mice with ABCA1 loss or decreased expression are more susceptible to worsened vascular endothelial injury, stroke, cerebral ischemia reperfusion injury, and neurological diseases such as Alzheimer’s disease (51–53). Hexb has also been found to affect the immune function of microglia and astrocytes in Sandhoff disease model mice and schizophrenia patients (54–56).

In addition, they also affect known thalamic haemorrhage-regulating genes. We constructed a map of the significant SNP sites of Apoe, Abca1 and Hexb, the miRNA−mRNA regulatory network and the motif binding domain and made a relatively complete annotation of the CPSP pathogenesis involving these key genes.

This study has some limitations. First, the source of sequencing samples was mice rather than human patients, which cannot perfectly reflect the clinical characteristics of CPSP, and there may be differences in the molecular characteristics. Second, we lacked animal experiments that verified the identified key genes and their mechanisms. In future research, we will focus on proving the correlation between Apoe, Abca1 and Hexb and CPSP and further explore their upstream and downstream pathways and signaling targets in neurons.

In summary, we identified three key immune-associated genes, namely, Apoe, Abca1, and Hexb, which can be used as potential genetic biomarkers for CPSP prediction and treatment. Additionally, we provided insights into the mechanisms of CPSP development at the transcriptome level and performed corresponding miRNA and SNP site predictions.

## Data availability statement

The datasets presented in this study can be found in online repositories. The names of the repository/repositories and accession number(s) can be found below: GSE227033 (GEO).

## Author contributions

TH: Conceptualization, methodology, experiments, software, and formal analysis. YX: Methodology, experiments, software and experiments; YZ: Validation and formal analysis; YG: Writing - review & editing and resources; JG: Writing - review & editing, visualization, project administration, and funding acquisition. All the authors read, discussed, and agreed to the final manuscript.

## Supplementary material

Supplementary Figure 1 Thalamic haemorrhage produces pain hypersensitivity. The microinjection of collagenase IV (Coll IV) into the ventral posterior medial nuclei and ventral posterior lateral nuclei resulted in an increased frequency of paw withdrawal in response to 0.07 g **(A)** and 0.4 g **(B)** von Frey filaments and a decreased latency of paw withdrawal in response to thermal **(C)** and cold **(D)** stimuli on the contralateral side. There were no observed alterations in paw withdrawal frequencies **(E, F)** and latency **(G)** on the ipsilateral side. n = 10 mice per group. *P < 0.05 versus the saline-treated group at the corresponding time points.

Supplementary Figure 2 Biology function Enrichment analysis of key genes. The left is the GO analysis, and the right is the KEGG analysis.

Supplementary Figure 3 Pearson correlation heatmap of key genes and immune factors. **(A–E)** represent chemokines, immunoinhibitors, immunostimulators, MHCs, and receptors, respectively.

Supplementary Figure 4 Motif transcriptional regulation analysis. **(A)** The distribution of AUC values ​​of the enriched motifs, which was calculated from the recovery curves of key gene-pair motif rankings. **(B)** Three motifs with higher AUC. In the figure, the red line is the mean value of each motif recovery curve, the green line is the mean ± SD, and the blue line is the current motif recovery curve. **(C)** The miRNA network of key genes; blue indicates mRNA, and orange indicates miRNA.

Supplementary Figure 5 Analysis of regulatory genes in thalamic haemorrhage disease. **(A)** Differences in the expression of disease-regulated genes. Blue indicates control patients, and pink indicates disease patients. **(B)** Pearson correlation analysis of key genes and disease genes. Blue indicates a negative correlation, and red indicates a positive correlation.

Appendix 1 Marker genes specific to cell subtypes.

Appendix 2 Significant SNP sites of key genes.
