# Supplementary figures and images for: Combination of single-nucleus and bulk RNA-seq reveals the molecular mechanism of thalamus haemorrhage-induced central poststroke pain
Source: Front Immunol. 2023 Apr 20;14:1174008. doi: 10.3389/fimmu.2023.1174008 (PMC10157064; doi:10.3389/fimmu.2023.1174008)

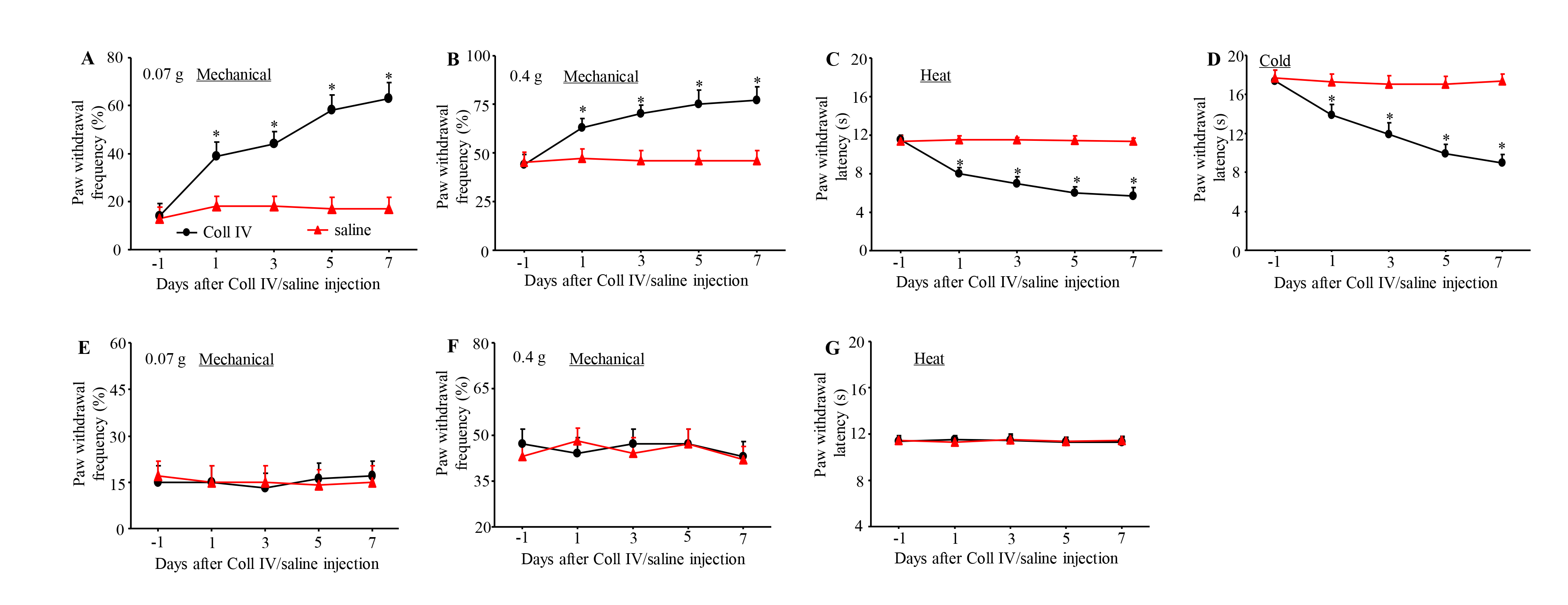

Supplement: Supplementary Figure 1 — Thalamic haemorrhage produces pain hypersensitivity. The microinjection of collagenase IV (Coll IV) into the ventral posterior medial nuclei and ventral posterior lateral nuclei resulted in an increased frequency of paw withdrawal in response to 0.07 g (A) and 0.4 g (B) von Frey filaments and a decreased latency of paw withdrawal in response to thermal (C) and cold (D) stimuli on the contralateral side. There were no observed alterations in paw withdrawal frequencies (E, F) and latency (G) on the ipsilateral side. n = 10 mice per group. *P < 0.05 versus the saline-treated group at the corresponding time points. [file Image_1.tif]

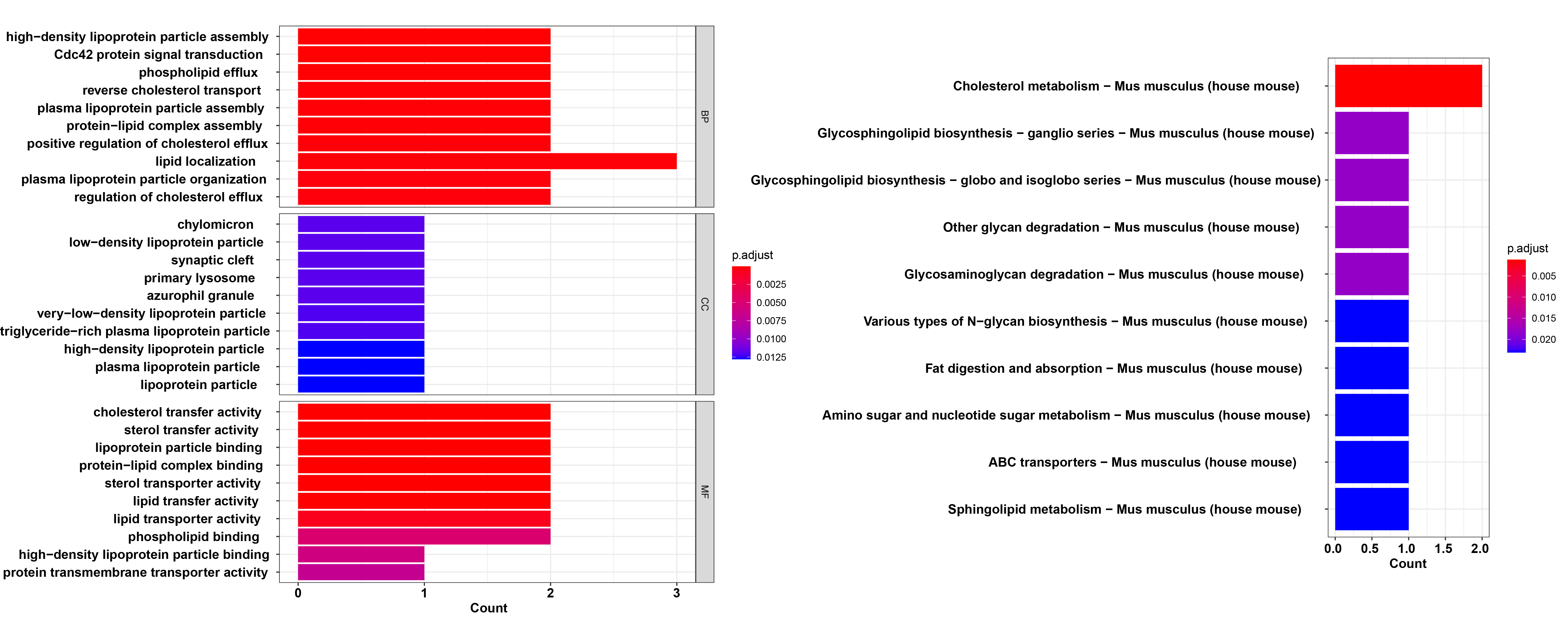

Supplement: Supplementary Figure 2 — Biology function Enrichment analysis of key genes. The left is the GO analysis, and the right is the KEGG analysis. [file Image_2.tif]

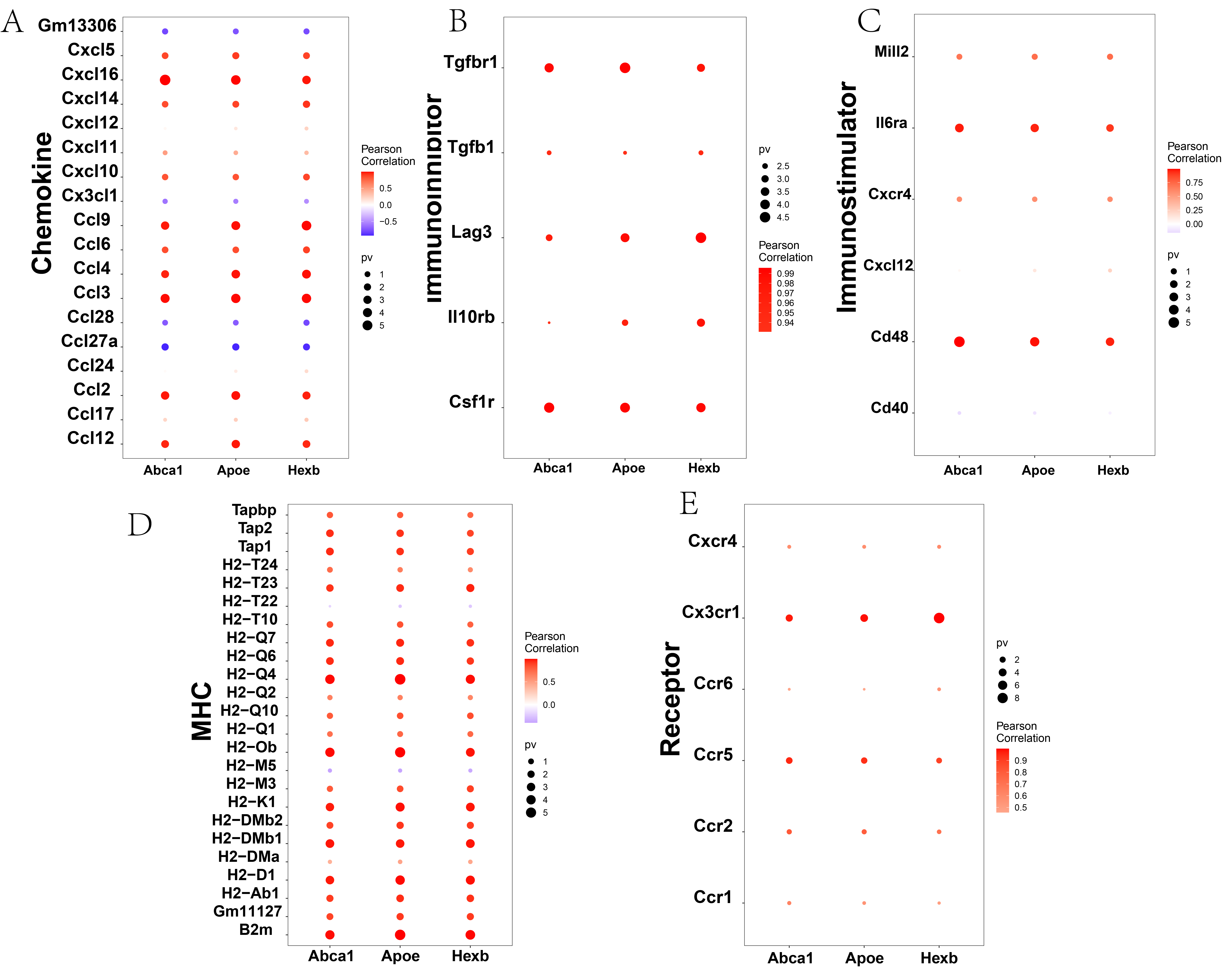

Supplement: Supplementary Figure 3 — Pearson correlation heatmap of key genes and immune factors. (A–E) represent chemokines, immunoinhibitors, immunostimulators, MHCs, and receptors, respectively. [file Image_3.tif]

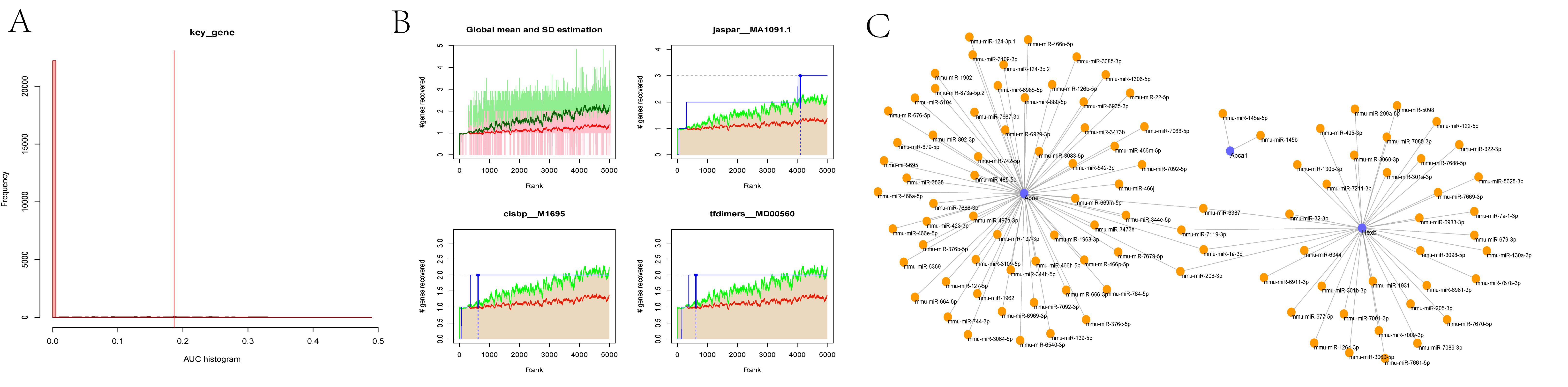

Supplement: Supplementary Figure 4 — Motif transcriptional regulation analysis. (A) The distribution of AUC values ​​of the enriched motifs, which was calculated from the recovery curves of key gene-pair motif rankings. (B) Three motifs with higher AUC. In the figure, the red line is the mean value of each motif recovery curve, the green line is the mean ± SD, and the blue line is the current motif recovery curve. (C) The miRNA network of key genes; blue indicates mRNA, and orange indicates miRNA. [file Image_4.jpeg]

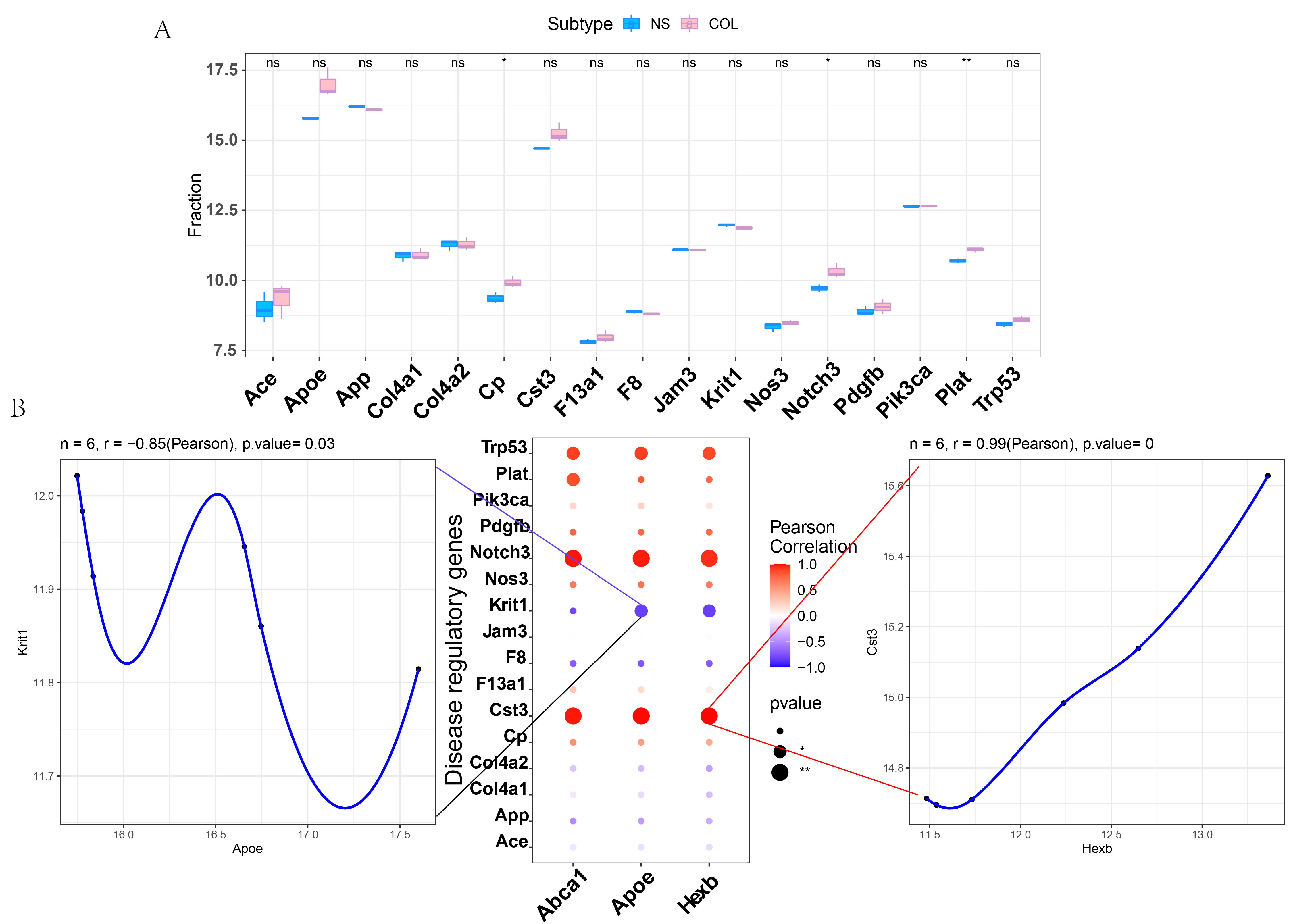

Supplement: Supplementary Figure 5 — Analysis of regulatory genes in thalamic haemorrhage disease. (A) Differences in the expression of disease-regulated genes. Blue indicates control patients, and pink indicates disease patients. (B) Pearson correlation analysis of key genes and disease genes. Blue indicates a negative correlation, and red indicates a positive correlation. [file Image_5.tif]
